# Novel Fatty Acid Biomarkers in Psoriasis and the Role of Modifiable Factors: Results from the METHAP Clinical Study

**DOI:** 10.3390/biom14091114

**Published:** 2024-09-04

**Authors:** Evangelia Sarandi, Sabine Krueger-Krasagakis, Dimitris Tsoukalas, George Evangelou, Maria Sifaki, Michael Kyriakakis, Efstathia Paramera, Evangelos Papakonstantinou, Gottfried Rudofsky, Aristides Tsatsakis

**Affiliations:** 1 Laboratory of Toxicology and Forensic Sciences, Medical School, University of Crete, 71003 Heraklion, Greece; 2 Metabolomic Medicine, Health Clinics for Autoimmune and Chronic Diseases, 10674 Athens, Greece; research@metabolomicmedicine.com; 3 Dermatology Department, University Hospital of Heraklion, 71110 Heraklion, Greece; 4 European Institute of Molecular Medicine, 00198 Rome, Italy; 5 Research Group of Clinical Pharmacology and Pharmacogenomics, Faculty of Pharmacy, School of Health Sciences, National and Kapodistrian University of Athens, 15701 Athens, Greece; 6 Neolab SA Medical Laboratory, 125 Michalakopoulou Street, 11527 Athens, Greece; 7 Clinic of Endocrinology and Metabolic Disorders, Cantonal Hospital Olten, 4600 Olten, Switzerland; gottfried.rudofsky@hin.ch

**Keywords:** psoriasis, lipid metabolism, network analysis, targeted metabolomics, biomarkers, modifiable factors

## Abstract

Psoriasis is a chronic, immune-mediated skin condition with significant metabolic complications. Although lipid metabolism is linked to its pathogenesis, reliable biomarkers and the impact of modifiable factors remain underexplored. The aim of the present study was to identify potential biomarkers, study the affected metabolic networks, and assess the role of dietary and lifestyle factors in psoriasis. Plasma samples from 56 patients with psoriasis and 49 healthy controls were analyzed, as part of the Metabolic Biomarkers in Hashimoto’s Thyroiditis and Psoriasis (METHAP) clinical trial. Using Gas Chromatography-Mass Spectrometry 23 fatty acids and their ratios were quantified, revealing significant changes in psoriasis. Specifically, lower levels of α-linoleic acid (C18:3n3), linoleic acid (C18:2n6), and gamma-linolenic acid (C18:3n6) were observed along with higher levels of eicosatrienoic acid (C20:3n3), eicosapentaenoic acid (C20:5n3), and erucic acid (C22:1n9). Total polyunsaturated fatty acids (PUFA) were significantly decreased, and the ratio of saturated to total fatty acids (SFA/Total) was increased in psoriasis (*p*-values < 0.0001). Linear regression identified α-linoleic acid, linoleic acid, eicosatrienoic acid, and eicosapentaenoic acid as potential biomarkers for psoriasis, adjusting for demographic, dietary, and lifestyle confounders. Network analysis revealed key contributors in the metabolic reprogramming of psoriasis. These findings highlight the association between psoriasis and fatty acid biomarkers of inflammation, insulin resistance and micronutrients deficiency, suggesting their potency in disease management.

## 1. Introduction

Psoriasis is an inflammatory chronic skin disease affecting 1–3% of the population. The most common type is plaque psoriasis, accounting for 90% of the cases, and its manifestation includes erythematosus patches of skin covered with silvery scales and well-defined borders. Psoriatic lesions can be itchy and painful and can coexist with psoriatic arthritis, having a deleterious impact on patients’ quality of life. Overall, psoriasis is responsible for 5.6 million all-age disability-adjusted life-years (DALYs), according to the Global Burden of Disease Study report [1,2].

The onset of psoriasis is multifactorial and occurs as a result of the combination of both genetic and non-genetic factors, including topical triggers such as a trauma or streptococcus infection, chemical-related such as drugs, vaccines, air pollutants, and smoking. Metabolism-related factors include alcohol consumption, micronutrient deficiencies, metabolic syndrome and mental stress. The mechanism through which external triggers lead to the irregular proliferation of keratinocytes and the progression to psoriatic lesions involves innate and adaptive immunity cells as discussed elsewhere [3,4]. Central to the etiopathogenesis of psoriasis is the metabolic reprogramming of involved cells, providing the energy and nutrients required for the inflammatory response and cellular metabolite interaction. Th-17 cells dominate skin psoriatic lesions, and previous studies have identified alterations in the metabolic pathways of T-cells, including fatty acid metabolism [5,6]. Fatty acids are structural components of cellular membranes, energy sources, and master regulators of inflammation processes, metabolic networks, and hormone production. The analysis of circulating fatty acids provides valuable insights into the dietary intake and endogenous metabolism of lipids while reflecting the metabolic state shaped by past exposures. Lipid metabolism studies have significantly contributed to the discovery of metabolic biomarkers as potential predictive tools and treatment targets in cardiometabolic and other chronic diseases [7,8,9]. Studies on psoriasis metabolic fingerprint have identified several alterations in biochemical pathways, even though the discovery of fatty acid biomarkers has been hampered by the small number of studies and group heterogeneity [10,11]. The aim of the present study was to assess psoriasis’ total plasma fatty acid levels and to investigate the effect of environmental factors on the disease-related metabolic imprint.

## 2. Materials and Methods

In this case-control study, patients with psoriasis and individuals with no diagnosed acute or chronic conditions (control group) were included based on the following criteria [12].

Inclusion criteria: Adults 18–60, male or female with normal BMI. Psoriatic disease was diagnosed by a specialized dermatologist and the PASI score and type of psoriasis were reported.

Exclusion criteria: Athletes, pregnant or lactating women, obese (BMI > 30). For the control group, individuals with acute or chronic disease or current infection or taking antidepressants or drugs were excluded. For the group of psoriasis, patients with severe comorbidities (cancer, type 2 diabetes, kidney or liver failure, coronary heart disease) were excluded.

Baseline data included age, sex, BMI, presence of comorbidities, medication, supplements, exercise, smoking, alcohol, and diet through the Mediterranean questionnaire MEDAS.

### 2.1. Ethics

This study is part of the registered clinical trial NCT04693936 (https://clinicaltrials.gov/study/NCT04693936, accessed on 5 August 2024) entitled Metabolic Biomarkers in Hashimoto’s Thyroiditis and Psoriasis (METHAP). Participants were informed of the details of the study and that full anonymization would be ensured according to the request of the EU General Data Protection Regulation (GDPR), and were requested to sign an informed consent after the details of the study were explained. The study has been approved by the Research Ethics Committee of the University of Crete (AP 147/10072020).

### 2.2. Sample Collection

Peripheral blood was collected from fasted participants in vacuum blood collection tubes with K2 ethylene diamino tetra-acetic acid EDTA and plasma was isolated through centrifuge at t 1500× *g* at 4 °C. Hemolyzed samples were discarded and patients were requested to repeat the blood collection.

Chemicals: The internal standard used for the plasma metabolomic analysis was methyl nonadecanoate (Supelco-Sigma-Aldrich, St. Louis, MO, USA) and the calibration of fatty acids was based on an FA methyl esters mix (Supelco-Sigma-Aldrich, St. Louis, MO, USA), which was used to calibrate the metabolites. Methanol, HCl, n-hexane (Merck KGaA, Darmstadt, Germany), and 2,6-i-tert-butyl-4-methylphenol (BHT) (Sigma-Aldrich, St. Louis, MO, USA) were also used in the fatty acid measurement.

### 2.3. Targeted Metabolomics

#### 2.3.1. Fatty Acids Extraction

The quantification of total plasma fatty acids was performed by adding 200 μL of the internal standard (methylnonadecanoate in hexane and BHT) to 100 μL of sample. In total, 5% *v*/*v* methanolic HCl was used to hydrolyze and derivatize the fatty acids to their methyl-esters (FAMEs) at 90 °C for 60 min. Following the incubation, samples were kept at room temperature and hexane was used to extract the FA methyl esters.

#### 2.3.2. Gas-Chromatography/Mass Spectrometry

The separation and detection of fatty acids were performed with the Agilent 7890A-5975C GC-MS (Agilent Technologies, Santa Clara, CA, USA), HP-5 ms capillary column (30 m × 250 µm × 0.25 µm) and helium was used for carrier gas. In total, 1 μL os sample was transferred to GC-MS injection vials and metabolites were separated using an oven temperature program as follows: initial temperature at 70 °C, followed by 290 °C for 4 min total time at a ramp rate of 4 °C/min. Fatty acid identification and quantification were performed using the Agilent Chemstation software (E.02.02.1431, Copyright 1989–2011, Agilent Technologies Inc.), where the acquisition of the MS was in the scan mode, and the standard solution was used to compare the retention time and mass spectra with that of the samples. The peak area of the samples and the standard solution ratio were used for the linear calibration curves and the fatty acids’ quantification.

#### 2.3.3. Statistical Analysis

Statistical analysis was performed using Excel (Microsoft Excel 2019), SPSS v22.0 (IBM Corp., Armonk, NY, USA), and Metaboanalyst v6.0 [13]. Data were scanned for missing values and outliers and graphically assessed for normality using QQplots. There were no cases with missing values or outliers for fatty acids values, and thus, all analyzed samples were included. Missing data on MEDAS score, alcohol, and exercise (<1%) were imputed by the mean values of the respective group. A Pearson chi-squared test was used to compare categorical variables, while a *t*-test (normal distribution) or Mann–Whitney test was conducted for continuous variables. Univariate analysis to test differences between means of independent samples was performed under the hypothesis (Hο) of non-difference for non-normal distributions. Enrichment analysis was performed using the concentration table of metabolite values against the Kyoto Encyclopedia Genes and Genomes (KEGG). Data were normalized, log-transformed, and Pareto scaled for MSEA and Network analysis on MetaboAnalyst. All statistical tests were two-sided, and a *p*-value < 0.05 was considered statistically significant.

## 3. Results

In the present study, 105 participants were analyzed, including 56 patients with plaque psoriasis and 49 age- and gender-matched individuals in the control group. The population characteristics are shown in Table 1. The two groups were similar in age, sex, and dietary and lifestyle habits, as assessed by the Mediterranean Diet Score (MEDAS), while mean BMI levels were higher in the PSO group, even though only normal-weight patients were included (25.6 ± 3.3 compared to 23.9 ± 2.5, *p* = 0.006). Alcohol consumption was comparable between the two groups, while exercise levels were statistically significantly lower in the psoriasis group (60.9% of patients did not exercise compared to 24.5% of control). Within the psoriasis group, more than half of the patients (66.1%) were not receiving systemic treatment, and the mean PASI score was 7.4.

### 3.1. Total Fatty Acids Profiling in Psoriasis

**Figure 1:**
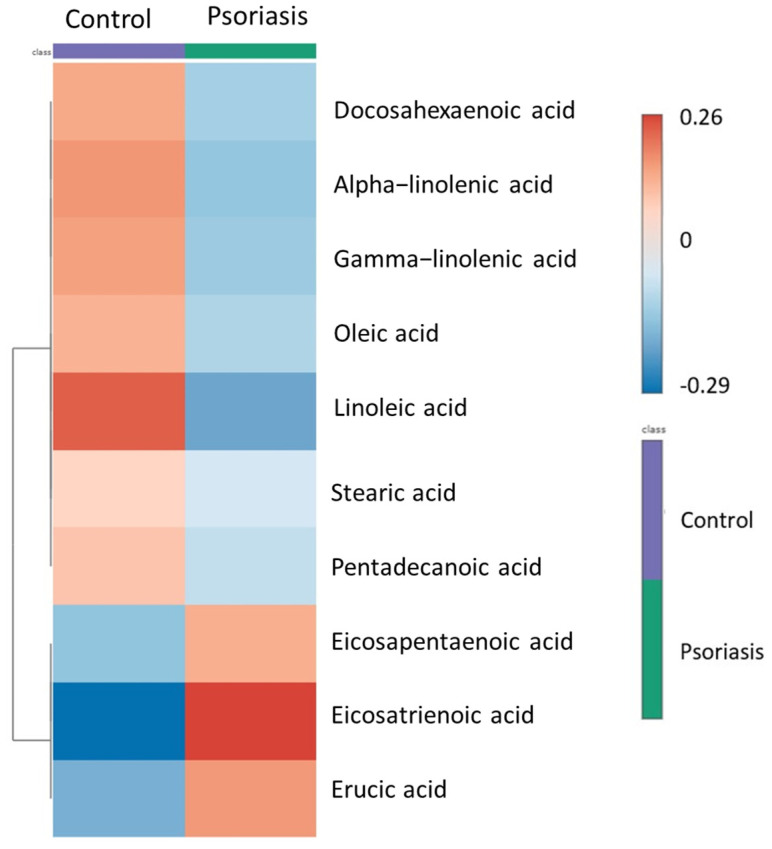
Hierarchical clustering heat map of the most significantly altered fatty acids in psoriasis compared to control (top 10) based on *t*-test/ANOVA: the color-coded cells indicate the metabolite concentration ranging from high (red) to low (blue). Docosahexaenoic, C22:n3; Alpha−linolenic acid, C18:3n3; Gamma−linolenic acid, C18:3n6; Oleic acid, C18:1ω9 cis; Linoleic acid, C18:2ω6; Stearic acid, C18:0; Pentadecanoic acid, C15:0; Eicosapentaenoic acid, C20:5ω3; Eicosatrienoic acid, C20:3ω3; Erucic acid, C22:1ω9.

A total of 36 comparisons were performed, including 23 total plasma fatty acids and 12 ratios—between 56 patients with psoriasis and 49 individuals in the control group. Table 2 shows the measured fatty acids’ concentrations (mean ± SD) and their ratios and totals. The estimation of the total for each group (e.g., PUFA) was performed using the sum of the measured fatty acids for each category. After correcting for multiple comparisons using the Bonferroni method, four metabolites, three from the PUFA (C18:3 n3, C20:3 n3, C18:2 n6) and one from the MUFA group (C22:1 n9), were found to be significantly different between the two groups (*p* < 0.002) and six additional metabolites were altered at a nominal significance (*p* < 0.05) (C20:5 n3, C22:6 n3, C18:3 n6, C18:1 n9 cis, C18:0, C14:1). In the group of ratio totals, SFA/Total FA, PUFA, and Total FA were statistically significantly different after the Bonferroni correction, while AA/EPA, Total omega-6/Total Omega-3, Long chain/Short + Very Long chain, and MUFA reached nominal significance. A heat map was constructed using hierarchical clustering to visualize the relative differences in the concentrations between the groups (Figure 1). Due to the significantly different levels of total fatty acids between the two groups, non-parametric tests were applied in the total plasma fatty acids composition expressed as a percentage of fatty acids compared to the total fatty acids (Supplementary Table S1). Additional markers were identified after the Bonferroni corrections, including C205n:3, C16:0, C24:0, and C14:1.

### 3.2. Correlation of Total Plasma Fatty Acids with Population Characteristics

A Spearman correlation analysis was performed to investigate the dependence of total plasma fatty acid levels on dietary and lifestyle factors in psoriasis. Table 3 shows the statistically significant correlations in bold and their *p*-values. Overall, the observed associations were weak to moderate (correlation coefficient ≤ 0.5). The strongest associations were observed between exercise and C20:5n3 (r = 0.5609, *p* < 0.0001), while there was an inverse correlation with the omega-6/omega-3 ratio (r = −0.484, *p* = 0.001).

No correlations were detected between the MEDAS score and plasma fatty acids, and alcohol consumption was positively correlated with C20:3n6 (r = 0.281, *p* = 0.036). The type of treatment was negatively correlated with C20:3n3, C20:3n6, C24:1n9, C20:0, and C22:0 and positively associated with C18:1n9 cis, and the associations were weak to moderate (correlation coefficient < 0.5). Nested analysis on patients receiving systemic treatment (biological therapy or oral immunosuppressants) (*n* = 19) revealed a negative association with C18:2n6 (r = −0.537, *p* = 0.018) for patients under immunosuppressants compared to biological treatment. Smoking was moderately linked to C20:4 n6, C16:0, C18:0, SFA, PUFA, and TOTAL fatty acids (correlation coefficient r < 0.5). Positive associations were detected between BMI and C20:3 n6, C16:1 n7, C18:1 n9 cis, C12:0, and Total fatty acids. Age and gender were mildly associated with C18:3 n6, C18:1 n9 cis, C20:1 n9, C22:0, Total, and MUFA. Correlation analysis of fatty acids changes and the PASI score showed a moderate positive association for C18:0 and C16:0 in 33 cases with available disease stage data.

Total plasma fatty acids that were statistically significantly changed in patients with psoriasis were further explored with linear regression analysis to investigate the predictive ability of C18:3n3, C20:3n3, C20:5n3, C18:3n6, C18:2n6, and C22:1n9, adjusting for confounders. The results of the regression are depicted in Table 4, and statistically significant associations are shown in bold. All models met the goodness of fit, normality, heteroscedacity (QQ plots), and autocorrelation criteria (F statistic and Durbin Watson statistic, respectively). On models tested, except for C22:1n9, the C18:3n3, C20:3n3, C20:5n3, C18:3n6, and C18:2n6 were significantly associated with psoriasis adjusting for gender, age, BMI, smoking, type of treatment, diet (MEDAS), alcohol consumption, and exercise. A stronger association was detected for C18:2n6 (F_9,95_ =5.274, *p* < 0.001) with acceptable Durbin–Watson independence of errors = 1.468, while in this model, no other associations with demographic and modifiable factors were observed.

### 3.3. Metabolic Pathways of Psoriasis

**Figure 2:**
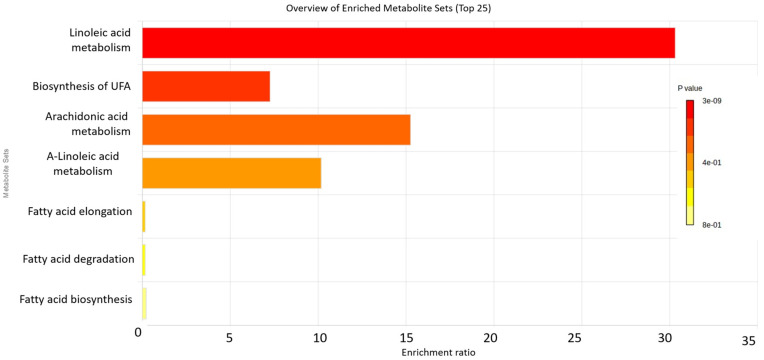
Metabolite set enrichment analysis (MSEA). Color-coded histogram representing the most affected metabolic pathways in plasma of patients with psoriasis compared to control depending on the number and importance of differentially expressed total fatty acids. UFA: Unsaturated Fatty acids.

**Figure 3:**
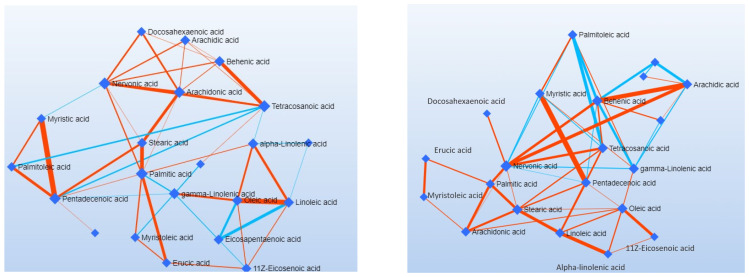
Debiased sparse partial correlation network analysis: left, control group; right, psoriasis group. Nodes represent the total plasma FA measured, and the color-coded edges represent the associations between the metabolites (red, positive; blue, negative). Thick lines indicate strong correlations.

To define the biological role of the metabolic changes in psoriasis, a metabolite set enrichment analysis (MSEA) was performed, and four metabolic pathways were found to be significantly affected (Figure 2). Linoleic acid (LA) metabolism was the most significantly affected pathway, followed by the biosynthesis of unsaturated fatty acids (UFA), arachidonic acid metabolism, and a-linolenic metabolism. The enrichment ratio depicted in the histogram of MSEA is calculated based on the ratio of differentially expressed metabolites by the expected hits of the specific metabolic pathway. Boxplots of the relative abundance of altered metabolites for each pathway are shown in Supplementary Figure S1. Pathways of fatty acids elongation, degradation, and biosynthesis were also altered. In addition, a network analysis was undertaken to explore changes in the relations between metabolites to consider possible non-canonical pathway changes in patients with psoriasis (Figure 3). Several metabolic connections (edges) were different between the two groups and the most important metabolites were identified. In the psoriasis group, nervonic acid had the most nodes (degree: 11; betweenness 63.48) compared to the control group indicating a central role to the other metabolites, followed by behenic acid (C22:0), oleic acid (C18:1n9cis), gamma-linolenic acid (C18:3n6), stearic (C18:0), and arachidic (C20:0). The full list of metabolites consisting of the networks and the values of degree and betweenness depicted in Figure 3 are shown in Supplementary Table S2.

## 4. Discussion

Fatty acid metabolism plays a key role in the development of psoriatic lesions due to the abundance of lipids and their derivatives ceramides, free fatty acids and cholesterol, in the skin. In addition, fatty acids regulate inflammatory responses through complex mechanisms, including the production of inflammatory mediator eicosanoids, the composition of the cell membrane, and several cellular functions [14]. A limited number of studies have analyzed the fatty acids profile of psoriasis, yielding insightful results. The aim of the present case-control study was to identify potential fatty acids biomarkers associated with psoriasis, to study the affected metabolic networks and define the role of dietary and lifestyle factors.

Metabolic profiling revealed significant differences including lower α-linoleic acid (C18:3n3), linoleic acid (C18:2n6), and gamma-linolenic acid (C18:3n6) and higher eicosatrienoic acid (C20:3n3), eicosapentaenoic acid (C20:5n3), and erucic acid (C22:1n9). Total polyunsaturated fatty acids (PUFA) were significantly decreased and the ratio of saturated to total fatty acids (SFA/Total) was increased in psoriasis. Among the tested metabolites, C18:3n3, C20:3n3, C20:5n3, C18:3n6, and C18:2n6 were significantly associated with PSO adjusting for gender, age, ΒMI, smoking, treatment, MEDAS, alcohol, and exercise and could serve as potent biomarkers. These findings are line with previous studies on serum and plasma showing abnormal levels of these fatty acids.

Specifically, Mysliwiec et al. compared the serum fatty acids of patients with psoriasis with healthy individuals and demonstrated decreased levels of C18:3n3, C18:2n6, and total n-3 PUFA in line with our results. On the other hand, C20:5n3 was increased in our group of patients, possibly explaining the lower levels of omega-6/omega-3 compared to the control group, in contrast to other studies [15,16]. In addition, Mysliwiec et al, reported higher SFA/UFA ratios, associated with obesity and these findings were also comparable to a later study by the same authors who compared circulating levels of fatty acids in psoriasis, psoriatic arthritis, and control. Recently, Marchlewicz et al. et al. analyzed the fatty acids content of erythrocytes of patients with psoriasis and identified that palmitoleic acid (C16:1) and stearic acid (C18:0) were the most abundant fatty acids while showing changed levels of specific fatty acids in relation to BMI levels, type of treatment, and disease severity [17].

Omega-3 and omega-6 polyunsaturated fatty acids are critical in the production of eicosanoids that mediate inflammatory responses, and their relative levels have been associated with psoriasis onset and disease severity [11,15,16]. In the present analysis, patients with psoriasis had lower levels of C18:3n3, C18:2n6, C18:3n6, and C22:6n3 but higher levels of C20:3n3 and C20:5n3. C18:2n6 accounts for 15% of the epidermis and, although it is exclusively obtained through diet, exerts an important role in the permeability and structure of the skin [18]. Similar roles have been observed in C18:3n6, C20:5n3, and C22:6n3 in the organization of the lipid membrane and other intra- and extracellular roles of cells [19]. A possible explanation for the decreased C182n:6 levels in the plasma of patients with psoriasis suggests mobilizing C18:2n6 from the periphery to the skin for its conversion to C20:4n6, which is known to accumulate in the skin [20]. Plasma levels of C20:4n6 (expressed as composition) were significantly increased in the group of psoriasis analyzed here (*p* = 0.0010), though higher than the Bonferroni threshold. The findings of the present study are in line with the observations of three other case-control studies that show reduced C182n:6 levels in psoriasis, and we further demonstrate that this association might be independent of demographic, dietary, and lifestyle factors [16,20,21].

An extensively studied role of omega-3 and omega-6 is their involvement in the production of inflammatory mediators, which is regulated by the relative abundance of the omega-3 to omega-6 ratio and the activity of the responsible desaturases [22,23]. Even though our study regarding the total omega-6/omega-3 yielded contradicting results of other studies, this could be due to the higher levels of C20:5n3 and C22:6n3 in the case group, even though C22:6n3 was lower. Higher levels of C20:5n3 but not C22:6n3 could be due to dysfunction of the C20:5n3 to C22:6n3 conversion catalyzed by desaturases and elongases. These enzymes are sensitive to micronutrient deficiencies and have been associated with insulin resistance, diabetes, and other chronic conditions [22,23]. In line with previous studies, plasma PUFA was lower, and the SFA/Total ratio was increased in patients with psoriasis compared to control, providing further evidence of their potential role in disease etiopathogenesis [16,24].

Analysis of MUFA identified C22:1n9 as statistically significantly different after the correction of multiple comparisons. C22:1n9 has not been reported in psoriasis even though it is known for its potential role in lipid dysregulation-associated health effects when consumed in high doses, for example, through bakery product consumption. In addition, when fatty acids were analyzed as composition (%total fatty acids), C14:1 was statistically significantly higher in the psoriasis group. Further to SFA, the SFA/total ratio and the percentage of C16:0 and C24:0 were significantly higher, in line with previous studies [16,25]. Palmitic acid (C16:0) has been extensively studied for its pro-inflammatory role involving metabolic molecular mechanisms and it has been shown to enhance the inflammatory response of epidermal keratinocytes [26]. Data on lignoceric acid (C24:0) in psoriasis are limited, though its ceramide has been associated with a higher PASI score in obese patients [27].

Previous research on the impact of SFA on the inflammatory processes of psoriasis has identified a central role in metabolic syndrome, insulin resistance and obesity. Metabolic syndrome is the most common comorbidity of psoriasis affecting 30–40% of the cases [28]. However, dysregulated metabolic pathways in psoriasis are also apparent in non-obese individuals (BMI < 30) and it has been experimentally shown that dietary factors rather than the obese state are related to psoriasis severity [29,30,31]. In the present study, we analyzed fatty acid metabolism in non-obese patients and identified several metabolic alterations independent of BMI, which was further validated by linear regression analysis. In addition, exercise exerted a positive link with C20:5n3 (r = 0.489, *p* = 0.01) and C22:6n:3 (r = 0.309, *p* = 0.037) in the psoriasis group, even though the associations observed were relatively weak (<50%). Moderate physical activity is known for its beneficial effects in weight loss and improved metabolism through increased lipolysis in the adipose tissue and substrates utilization from the skeletal muscle. There is some evidence of its beneficial effects on psoriasis, and further research is focused on the potential clinical benefit [32]. It has been suggested that psoriasis and exercise have a bidirectional relationship where psoriasis severity might affect the levels of exercise due to discomfort and psychological reasons, while on the other hand, exercise may improve the inflammatory status regardless of obesity [33]. In the present study, patients with psoriasis exercised significantly less compared to the control group, even though it did not affect the fatty acid differences between groups. Further studies could explore the impact of exercise on fatty acids metabolism and psoriasis phenotype considering confounders such as stage of disease, BMI, diet, educational and socioeconomic status, and age.

A strength of the present case-control study is the quantification of 23 distinct fatty acids to characterize the metabolic profile of psoriasis in a relatively homogenous population of normal BMI patients without any severe comorbidities (type 2 diabetes, non-alcoholic fatty liver disease, cardiovascular disease, etc.). Most metabolomics studies employ exploratory methods, such as NMR, analyze specific small subsets of metabolites for their absolute quantification. Also, their aim is to develop predictive tools that narrow down the variables into one or two variables using PCA or similar methods. In psoriasis, studies on fatty acids are limited and mostly focused on disease severity and comorbidities (obesity, metabolic syndrome), while they do not consider dietary and lifestyle factors. Therefore, this is the first study to explore the association of total plasma fatty acids with psoriasis considering dietary and lifestyle factors. Here, we identified C18:3n:3, C20:3n3, C20:5n3, C18:3n6, and C18:2n6 associated with the presence of psoriasis after adjustment of demographic and lifestyle factors, suggesting their potency as biomarkers employed by future studies. A secondary aim of the present study was to show the full dysregulation of fatty acid metabolism by employing pathway and network analysis. DSPC network analysis offers the advantage of looking for metabolite–metabolite interactions between two groups and identifying possible indirect non-canonical associations forming new research questions. In this study, we identified nervonic acid (C241n:9), behenic acid (C22:0), oleic acid (C18:1n9cis), gamma-linolenic acid (C18:3n6), stearic acid (C18:0), and arachidic (C20:0) (Figure 3). Nervonic acid, a monounsaturated fatty acid, is commonly found at high levels in neurological disease due to its abundance in myelin-covered cells. However, nervonic acid biosynthesis indicates a potential role in insulin resistance or hyperinsulinemia, which is common in psoriasis. In a case-control study, it was shown that nervonic acid might be associated with metabolic syndrome through plasmalogen synthesis steps, a pathway that was significantly enriched in patients with psoriasis. Further animal studies demonstrated that changes in nervonic acid might regulate energy and lipid metabolism [34,35].

The present study has certain limitations requiring the interpretation of these findings with caution. Dietary and lifestyle factors play a major role in metabolite absolute and relative levels. More detailed food questionnaires are required to determine the intake of fatty acid levels. The MEDAS score is a widely used validated questionnaire to estimate the Mediterranean score. However, inter-individual dietary habits may differentiate regarding the portion, the type of a certain food and the combination of nutrients, which can affect the fatty acids profile. More detailed and descriptive questionnaires would elucidate the potential role of dietary intake in fatty acid levels. Another limitation of this study is the lack of routine blood and biochemical tests that would identify potential clusters within groups or exclude participants who have abnormal biochemical measurements. Even though the current study did not assess the levels of inflammation markers, future studies could also explore the potential role of changes in α-linoleic acid, linoleic acid, eicosatrienoic acid, and eicosapentaenoic acid on the inflammatory phenotype of patients with psoriasis. Psoriasis is a T-cell-mediated disease involving the IL-17/IL-23 axis, and research has demonstrated a protective role of omega-3 fatty acids as anti-inflammatory mediators on the T-cell responses. Experimental studies on psoriatic mouse models and 3D skin models, show that omega-3 fatty acids administration leads to the downregulation of IL-17 and upregulation of anti-inflammatory regulatory T-cells [36,37]. In addition, Guo et al. showed that the fatty acids changes observed in patients with psoriasis were affected by anti-IL17 treatment potentially correlating with IL-17 levels [24]. Our analysis demonstrated that the fatty acids were associated with psoriasis, adjusting for confounders. The BMI was significantly higher in patients with psoriasis than the control and even though only non-obese participants were included, a subgroup analysis using a larger sample would clarify the role of BMI levels in the detected associations.

## 5. Conclusions

Psoriasis is an inflammatory skin disorder where lipid metabolism plays a central role to disease etiopathogenesis and metabolic comorbidities. The present case-control study demonstrated key metabolic changes in the plasma of patients with psoriasis highlighting an association between psoriasis and fatty acids biomarkers linked to inflammation, insulin resistance and micronutrient deficiencies. A- linoleic acid, linoleic acid, eicosatrienoic acid and eicosapentaenoic acid were identified as potential biomarkers while network and pathway analysis revealed further metabolic dysregulations. Overall, targeting fatty acids can increase our understanding of psoriasis’ complex mechanisms and contribute to the development of improved diagnostic and management strategies.

## Figures and Tables

**Table 1:** Population characteristics of patients with psoriasis and the control group.

	Cases (*n* = 56)	Control (*n* = 49)	*p*-Value
**Gender**			
**Female *n* (%)**	26 (46.4)	28 (57.1)	0.273 a
**Male *n* (%)**	30 (53.6)	21 (42.9)	
**Age (mean ±SD)**	41.8 ± 13.8	42.4 ± 8.9	0.825 b
**BMI (mean ± SD)**	25.6 ± 3.3	23.9 ± 2.5	**0.006** b
**Alcohol consumption**			
No alcohol *n* (%)	18 (39.1%)	13 (26.5%)	0.349 a
Up to 3 glasses per week *n* (%)	19 (41.3%)	27 (55.1)%	
Over 3 glasses/per week *n* (%)	9 (19.6%)	9 (18.4%)	
**Exercise**			
No exercise	28 (60.9%)	12 (24.5%)	**0.002** a
Up to 3 times per week *n* (%)	11 (23.9%)	24 (49%)	
Over 3 times per week *n* (%)	7 (15.2%)	13 (26.5%)	
**Smoking status *n* (%)**	29 (52.7%)	21 (42.9%)	0.315 a
**MEDAS (mean ± SD)**	6.3 ± 2.0	5.9 ± 1.9	0.494 a
**No Treatment**	26 (46.4%)		
**Topical cream**	11 (19.6%)		
**Immunosuppressants**	6 (10.7%)		
**Biological treatment**	13 (23.2%)		

*p*-values were estimated with Pearson chi-square (a) or non-parametric Mann–Whitney test due to non-normal data distribution for age and BMI (b). BMI, Body Mass Index; MEDAS, Mediterranean Diet Score. Values in bold indicate *p* < 0.05.

**Table 2:** Descriptive statistics of plasma total fatty acids (μmol/L), ratios, and totals in patients with psoriasis compared to the control group.

Fatty Acids	Case *n* = 56				Control *n* = 49				*p*-Value
	Min	Mean ± SD	Median	Max	Min	Mean+ SD	Median	Max	
**C18:3 n3**	**4.36**	**35.65 ± 16.11**	**36.77**	**93.46**	**5.23**	**51.21 ± 21.19**	**49.00**	**113.49**	**<0.0001**
**C20:3 n3**	**0.91**	**3.69 ± 10.21**	**2.00**	**78.15**	**0.07**	**1.48 ± 1.34**	**1.00**	**6.63**	**<0.0001**
C20:5 n3	10.1	67.44 ± 45.09	56.52	188.36	4.42	45.24 ± 25.37	41.97	121.66	0.021
C22:6 n3	37.62	113.61 ± 56.51	102.22	245.00	41.02	145.80 ± 56.74	138.65	250.32	0.003
C18:3 n6	4.27	29.32 ± 17.76	26.82	72.00	6.31	42.17 ± 27.48	40.3	124.94	0.018
**C18:2 n6**	**580.58**	**1830.31 ± 671.58**	**1816.03**	**4852.00**	**778.61**	**2814.83 ± 907.89**	**2998.49**	**4852.00**	**<0.0001**
C20:4 n6	219.27	424.04 ± 141.57	406.37	732.9	238.21	425.02 ± 132.03	405.06	855.15	0.86
C20:3 n6	2.16	82.57 ± 35.85	83.23	142.67	21.5	90.90 ± 45.76	84.22	269.56	0.644
C15:1	1.00	23.83 ± 20.45	18.74	99.44	6.7	22.20 ± 10.70	19.4	57.56	0.540
C16:1 n7	3.14	101.77 ± 75.52	86.88	485.37	23.16	85.91 ± 46.55	71.71	272.09	0.289
C18:1 n9 cis	564.03	1752.49 ± 637.26	1774.15	2922.67	625.08	2168.71 ± 672.51	2022.00	3489.00	0.003
C20:1 n9	1.47	15.40 ± 9.57	19.59	40.61	1.00	18.32 ± 10.58	18.14	45.42	0.320
**C22:1 n9**	**0.33**	**3.37 ± 9.52**	**2.00**	**7.61**	**0.1**	**1.30 ± 1.03**	**1.00**	**7.05**	**<0.0001**
C24:1 n9	22.26	59.60 ± 19.75	58.15	102.68	33.75	56.82 ± 14.72	55.21	93.92	0.411
C12:0	0,00	3.10 ± 2. 47	2.81	8.01	0.00	2.29 ± 2.35	2.2	11.05	0.97
C14:0	11.34	49.16 ± 30.92	40.44	140.00	14.4	41.75 ± 20.05	38.25	107.82	0.506
C15:0	1.57	5.76 ± 3.02	5.04	14.65	2.65	5.51 ± 1.84	5.17	10.55	0.686
C16:0	867.77	1919.41 ± 592.31	1866.71	3956.00	1193.93	1859.76 ± 401.04	1797.71	3027.04	0.658
C18:0	258.72	485.78 ± 137.83	469.86	922.97	312.27	541.64 ± 147.76	529.03	1030.33	0.024
C20:0	1.36	12.96 ± 7.20	11.15	32.37	0.00	13.86 ± 4.27	14.05	21.45	0.151
C22:0	10.94	37.30 ± 17.95	35.67	85.35	5.00	34.04 ± 10.93	33.2	63.69	0.57
C24:0	18.41	53.00 ± 16.76	51.21	89.78	25.00	50.46 ± 14.39	49.84	75.00	0.431
C14:1	0.11	2.17 ± 1.39	2.00	8.79	0.61	1.87 ± 1.56	1.5	8.43	0.007
**Ratios- Total**									
AA/DGLA	1.93	11.26 ± 36.73	4.91	278.4	2.19	5.41 ± 2.48	5.04	17.07	0.842
AA/EPA	2.00	9.50 ± 6.52	6.76	32.37	3.32	13.84 ± 14.13	10.35	77.22	0.013
Omega-6/Omega-3	5.3	11.83 ± 3.85	11.48	22.04	4.5	14.63 ± 5.31	13.14	30.47	0.004
Myristoleic/Myristic	0.0012	0.06 ± 0.08	0.05	0.55	0.02	0.05 ± 0.04	0.04	0.3	0.175
Palmitoleic/Palmitic	0.0019	0.05 ± 0.03	0.05	0.19	0.02	0.04 ± 0.02	0.04	0.09	0.286
Oleic/Stearic	1.48	3.72 ± 1.39	3.6	7.97	1.62	4.12 ± 1.36	4.06	7.81	0.114
**SFA/TOTAL**	**0.26**	**0.36 ± 0.07**	**0.35**	**0.52**	**0.23**	**0.31 ± 0.06**	**0.29**	**0.47**	**<0.0001**
L/S + VL SFA	9.43	17.56 ± 4.28	16.78	31.55	11.52	19.87 ± 5.19	19.3	30.59	0.035
SFA	1416.99	2563.70 ± 719.25	2501.61	5091.98	1758.91	2545.69 ± 545.26	2415.93	4168.91	0.883
**PUFA**	**1015.09**	**2586.67 ± 824.69**	**2472.3**	**6022.5**	**1300.66**	**3616.68 ± 1004.49**	**3614.78**	**5849.34**	**<0.0001**
MUFA	689.96	1958.65 ± 700.48	1922.51	3511.83	753.82	2355.15 ± 713.12	2246.32	3858.14	0.008
**TOTAL**	**4187.95**	**7108.74 ± 1840.70**	**6956.43**	**12,553.07**	**3895.32**	**8517.53 ± 1981. 35**	**8402.98**	**13,706.48**	**<0.0001**

*p*-values calculated with Mann–Whitney test. Bold values show *p*-values below the threshold after Bonferroni correction 0.05/23.

**Table 3:** Correlations between demographic and modifiable factors and total plasma fatty acids in psoriasis.

		Age	Gender	ΒΜΙ	Smoking Status	Treatment	MEDAS	Alcohol	Exercise	PASI
**C18:3 n3**	Corr. coeff.	−0.195	0.254	−0.148	−0.010	0.180	−0.059	0.052	0.080	−0.247
	*p*-value	0.150	0.058	0.277	0.942	0.185	0.667	0.702	0.559	0.181
**C20:3 n3**	Corr. coeff.	0.221	−0.344 **	−0.120	−0.062	−0.431 **	0.162	−0.098	−0.041	−0.027
	*p*-value	0.101	0.009	0.377	0.649	0.001	0.234	0.470	0.764	0.884
**C20:5 n3**	Corr. coeff.	−0.156	0.227	0.186	0.196	0.175	−0.188	−0.074	0.560 **	0.234
	*p*-value	0.250	0.092	0.169	0.148	0.196	0.166	0.586	0.000	0.205
**C22:6 n3**	Corr. coeff.	0.000	0.021	0.098	0.140	−0.030	0.031	0.192	0.441 **	0.165
	*p*-value	0.999	0.880	0.470	0.302	0.826	0.819	0.156	0.001	0.376
**C18:3 n6**	Corr. coeff.	0.352 **	0.328 *	0.242	0.223	0.159	−0.234	0.012	0.072	0.078
	*p*-value	0.008	0.013	0.072	0.098	0.241	0.082	0.929	0.599	0.678
**C18:2 n6**	Corr. coeff.	−0.076	−0.098	0.014	0.207	−0.118	−0.016	−0.041	0.237	0.054
	*p*-value	0.577	0.472	0.920	0.126	0.388	0.905	0.764	0.079	0.771
**C20:4 n6**	Corr. coeff.	−0.022	0.125	0.184	0.374 **	0.136	−0.188	0.086	0.164	0.131
	*p*-value	0.871	0.358	0.175	0.005	0.317	0.165	0.531	0.227	0.481
**C20:3 n6**	Corr. coeff.	−0.251	0.196	0.302 *	0.109	−0.265 *	−0.005	0.281 *	−0.008	0.195
	*p*-value	0.062	0.148	0.024	0.422	0.048	0.972	0.036	0.954	0.293
**C15:1**	Corr. coeff.	−0.242	0.054	0.078	0.109	−0.017	0.094	−0.013	0.210	0.282
	*p*-value	0.072	0.695	0.569	0.422	0.899	0.489	0.922	0.120	0.124
**C16:1 n7**	Corr. coeff.	−0.235	0.204	0.332 *	0.189	0.120	−0.176	0.017	0.185	0.036
	*p*-value	0.081	0.131	0.012	0.163	0.379	0.195	0.903	0.172	0.849
**C18:1 n9 cis**	Corr. coeff.	0.381 **	0.537 **	0.321 *	0.187	0.277 *	−0.198	0.168	0.246	−0.048
	*p*-value	0.004	0.000	0.016	0.168	0.039	0.143	0.216	0.068	0.796
**C20:1 n9**	Corr. coeff.	−0.304 *	0.383 **	0.042	0.182	−0.078	0.116	0.022	0.054	0.051
	*p*-value	0.023	0.004	0.760	0.179	0.569	0.395	0.870	0.692	0.786
**C22:1 n9**	Corr. coeff.	0.068	0.185	−0.004	0.170	0.126	−0.185	−0.014	−0.052	−0.038
	*p*-value	0.616	0.173	0.978	0.211	0.355	0.172	0.918	0.702	0.838
**C24:1 n9**	Corr. coeff.	0.190	−0.164	0.034	0.262	−0.269 *	0.038	0.118	0.314 *	0.173
	*p*-value	0.160	0.226	0.801	0.051	0.045	0.780	0.387	0.018	0.353
**C14:0**	Corr. coeff.	−0.236	0.209	0.138	0.124	−0.068	0.003	−0.129	0.125	0.208
	*p*-value	0.080	0.123	0.312	0.363	0.618	0.982	0.344	0.360	0.261
**C15:0**	Corr. coeff.	−0.032	−0.179	−0.027	0.156	−0.048	0.089	−0.088	0.188	0.184
	*p*-value	0.813	0.186	0.841	0.251	0.723	0.515	0.520	0.166	0.321
**C16:0**	Corr. coeff.	−0.083	0.118	0.214	0.289 *	−0.154	−0.032	−0.048	0.435 **	0.373 *
	*p*-value	0.544	0.388	0.113	0.031	0.256	0.816	0.727	0.001	0.039
**C18:0**	Corr. coeff.	−0.070	−0.123	0.200	0.264 *	−0.088	0.075	0.010	0.277 *	0.408 *
	*p*-value	0.610	0.368	0.140	0.049	0.521	0.581	0.942	0.039	0.023
**C20:0**	Corr. coeff.	0.192	−0.294 *	0.083	−0.140	−0.325 *	0.164	0.074	−0.064	0.219
	*p*-value	0.157	0.028	0.542	0.302	0.014	0.226	0.586	0.639	0.237
**C22:0**	Corr. coeff.	0.301 *	−0.365 **	−0.121	0.098	−0.410 **	0.222	−0.001	0.006	0.204
	*p*-value	0.024	0.006	0.375	0.471	0.002	0.100	0.994	0.967	0.272
**C24:0**	Corr. coeff.	−0.058	−0.093	0.162	0.255	−0.150	0.015	0.070	0.230	0.068
	*p*-value	0.672	0.496	0.232	0.057	0.270	0.913	0.608	0.088	0.716
**C14:1**	Corr. coeff.	0.048	0.133	0.131	0.182	0.210	−0.126	0.180	0.115	−0.008
	*p*-value	0.727	0.329	0.335	0.180	0.120	0.353	0.183	0.399	0.966
**C12:0**	Corr. coeff.	−0.056	−0.031	0.361 **	−0.008	−0.172	−0.116	−0.131	−0.004	0.107
	*p*-value	0.685	0.823	0.007	0.952	0.213	0.402	0.346	0.979	0.574
**SFA**	Corr. coeff	−0.097	0.076	0.210	0.313 *	−0.161	0.012	−0.026	0.439 **	0.359 *
	*p*-value	0.477	0.580	0.120	0.019	0.235	0.928	0.852	0.001	0.047
**PUFA**	Corr. coeff	−0.142	0.020	0.113	0.315 *	0.014	−0.083	0.039	0.302 *	0.124
	*p*-value	0.295	0.883	0.406	0.018	0.919	0.542	0.776	0.024	0.506
**TOTAL**	Corr. coeff	−0.273 *	0.270 *	0.268 *	0.342 **	0.053	−0.110	0.090	0.378 **	0.195
	*p*-value	0.042	0.044	0.046	0.010	0.698	0.419	0.509	0.004	0.292
**MUFA**	Corr. coeff	−0.394 **	0.530 **	0.332 *	0.200	0.256	−0.204	0.159	0.246	−0.018
	*p*-value	0.003	0.000	0.012	0.139	0.057	0.132	0.242	0.068	0.921
**Omega-6/Omega-3**	Corr. coeff	−0.021	−0.199	−0.125	−0.014	−0.205	0.008	0.019	−0.484 **	−0.094
	*p*-value	0.876	0.142	0.357	0.916	0.129	0.951	0.890	0.000	0.614

Spearman correlation coefficients. Associations that were statistically significant (* *p* < 0.05, ** *p* < 0.01, two-tailed) are shown in bold.

**Table 4:** Association of total plasma fatty acids and psoriasis adjusted for confounding factors.

	C18:3n3	C20:3n3	C20:5n3	C18:3n6	C18:2n6	C22:1n9
Constant	4.907 (3.636, 6.177)	0.880 (−1.257, 3.017)	3.316 (1.666, 4.966)	3.277 (1.666, 4.966)	8.129 (7.289, 8.969)	0.312 (−1.275, 1.899)
Group	**−0.406** (−0.68, 0.315) **	**1.046** (0.584, 1.508) ***	**0.473** (0.117, 0.831) **	**−0.447** (0.117, 0.831) **	**−0.343** (−0.525, −0.162) ***	0.271 (−0.073, 0.613)
Gender (F)	−0.3326 (−0.567, −0.084	0.034 (−0.373, 0.440)	−0.056 (−0.369, 0.258)	**−0.376** (−0.369, 0.258) **	−0.091 (−0.250, 0.069)	−0.015 (−0.317, 0.287)
Age	0.003 (−0.007, 0.013)	−0.015 (−0.032, 0.001)	0.007 (−0.006, 0.020)	**0.013** (−0.006, 0.020) **	−0.005 (−0.011, 0.002)	0.001 (−0.011, 0.014)
ΒMI	**−0.042** (−0.084, 0.000) *	−0.001 (−0.072, 0.070)	0.002 (−0.053, 0.057)	0.003 (−0.053, 0.057)	0.001 (−0.27, 0.029)	−0.008 (−0.061, 0.044)
Smoking	0.070 (−0.142, 0.283)	0.022 (−0.336, 0.380)	−0.081 (−0.357, 0.196)	0.237 (−0.357, 0.196)	0.081 (−0.059, 0.222)	0.241 (−0.025, 0.507)
Treatment	0.124 (−0.197, 0.445)	−0.385 (−0.926, 0.155)	0.169 (−0.248, 0.587)	0.135 (−0.248, 0.587)	−0.095 (−0.308, 0.117)	0.195 (−0.206, 0.597)
MEDAS	−0.002 (−0.060, 0.056)	−0.049 (−0.147, 0.048)	−0.050 (−0.125, 0.026)	−0.037 (−0.125, 0.026)	−0.029 (−0.068, 0.009)	0.021 (−0.052, 0.093)
Alcohol	−0.002 (−0.150, 0.146)	−0.058 (−0.308, 0.192)	−0.071 (−0.264, 0.121)	0.004 (−0.264, 0.121)	0.026 (−0.068, 0.009)	−0.165 (−0.350, 0.021)
Exercise	−0.063 (−0.031, 0.178)	0.048 (−0.358, 0.454)	**0.502** (0.188, 0.816) **	−0.048 (0.188, 0.816)	0.136 (−0.024, 0.295)	−0.202 (−0.504, 0.100)
R squared	0.195	0.254	0.209	0.245	0.333	0.167
Adjusted r squared	0.119	0.183	0.135	0.179	0.27	0.088
F statistic Sig. Change	0.011	0.001	0.006	0.001	0.000	0.035
Durbin-watson statistic	1.405	1.622	1.561	1.563	1.468	2.029

Linear regression analysis. Dependent variables were estimated in a natural log scale; Statistically significant associations are shown in bold, * *p* < 0.05, ** *p* < 0.01, *** *p* < 0.001; Group: Psoriasis.

## Data Availability

Dataset is available upon reasonable request from the corresponding authors.

## Supplementary Materials

The following supporting information can be downloaded at: https://www.mdpi.com/article/10.3390/biom14091114/s1, Figure S1: Boxplots of relative concentrations of metabolites participating in metabolic pathways of psoriasis; Table S1: Composition of total plasma fatty acids in psoriasis compared to healthy individuals (expressed as % total fatty acids). *p*-values calculated with Mann-Whitney test. Bold values show *p*-values below the threshold after Bonferroni correction 0.05/23; Table S2: Values of degree and betweenness centrality of the plasma fatty acids in psoriasis and healthy control group, used in the DSPC network analysis.
