# Supplementary material for: Novel Fatty Acid Biomarkers in Psoriasis and the Role of Modifiable Factors: Results from the METHAP Clinical Study
Source: Biomolecules. 2024 Sep 4;14(9):1114. doi: 10.3390/biom14091114 (PMC11430636; doi:10.3390/biom14091114)
Supplement: Supplementary file 1 [file biomolecules-14-01114-s001.zip › biomolecules-3168487-supplementary.pdf]

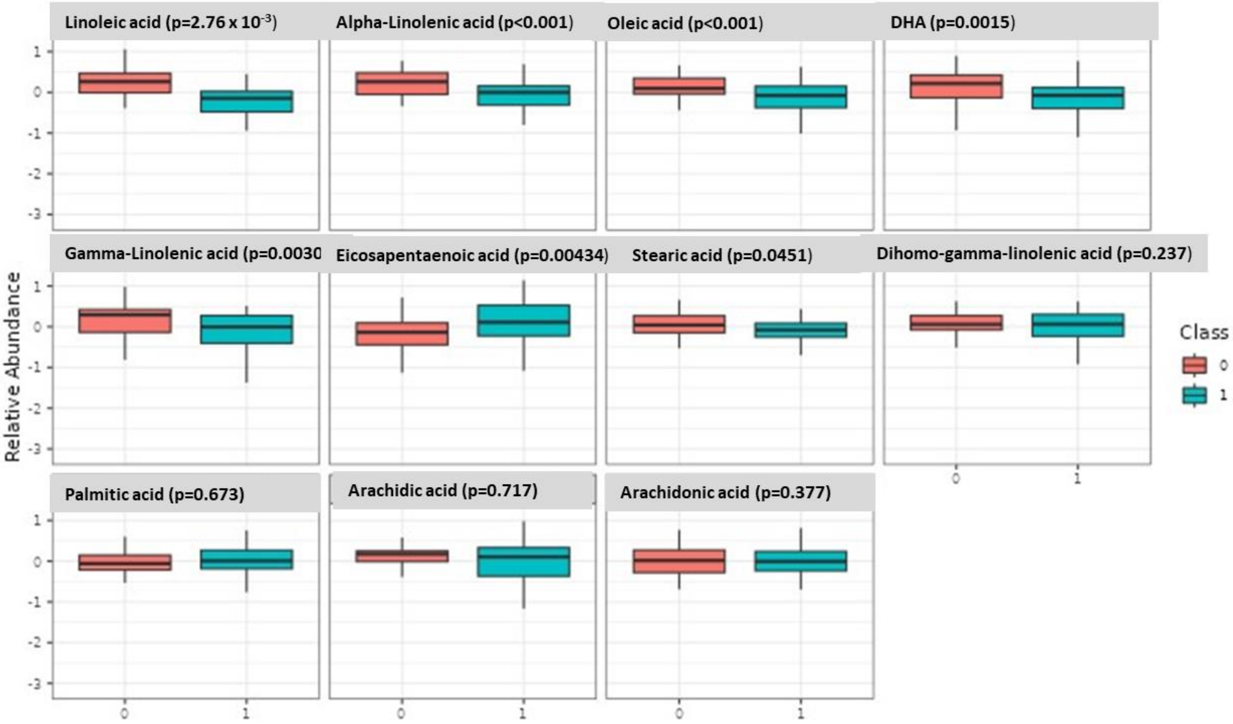


**Figure S1: Boxplots of relative concentrations of metabolites participating in metabolic pathways of psoriasis** Up: Alpha-Linolenic and Linoleic metabolism Down: Arachidonic acid metabolism. Metabolites of the control group are in red boxplots and of the psoriasis group are in green boxplots. DHA: Docosahexanoic acid

| GROUP | PSORIASIS | | | CONTROL | | | |
|---|---|---|---|---|---|---|---|
| | Mean | Median | Std. Deviation | Mean | Median | Std. Deviation | P-value |
| C18:3 n3 | 0,51 | 0,50 | 0,20 | 0,59 | 0,62 | 0,17 | 0,009 |
| **C20:3 n3** | **0,06** | **0,03** | **0,16** | **0,02** | **0,01** | **0,02** | **<0.0001** |
| **C20:5 n3** | **0,92** | **0,86** | **0,54** | **0,56** | **0,48** | **0,34** | **<0.0001** |
| C22:6 n3 | 1,58 | 1,49 | 0,62 | 1,77 | 1,75 | 0,71 | 0.148 |
| C18:3 n6 | 0,39 | 0,39 | 0,18 | 0,47 | 0,42 | 0,24 | 0.150 |
| **C18:2 n6** | **25,71** | **25,91** | **5,60** | **32,51** | **32,75** | **5,80** | **<0.0001** |
| C20:4 n6 | 5,98 | 5,74 | 1,36 | 5,09 | 4,91 | 1,37 | 0.001 |
| C20:3 n6 | 1,20 | 1,14 | 0,51 | 1,07 | 0,99 | 0,42 | 0.126 |
| **C14:1** | **0,03** | **0,03** | **0,03** | **0,02** | **0,02** | **0,02** | **<0.0001** |
| C15:1 | 0,32 | 0,29 | 0,23 | 0,26 | 0,25 | 0,09 | 0.326 |
| C16:1 n7 | 1,38 | 1,24 | 0,83 | 0,99 | 0,90 | 0,36 | 0.004 |
| C18:1 n9 cis | 24,46 | 24,09 | 6,24 | 25,11 | 24,52 | 4,18 | 0.616 |
| C20:1 n9 | 0,21 | 0,23 | 0,12 | 0,21 | 0,22 | 0,12 | 0.782 |
| **C22:1 n9** | **0,03** | **0,02** | **0,03** | **0,02** | **0,01** | **0,02** | **<0.0001** |
| C24:1 n9 | 0,86 | 0,78 | 0,30 | 0,70 | 0,64 | 0,22 | 0.001 |
| C12:0 | 0,04 | 0,04 | 0,04 | 0,03 | 0,02 | 0,03 | 0.057 |
| C14:0 | 0,67 | 0,60 | 0,33 | 0,49 | 0,46 | 0,18 | 0.003 |

| | | | | | | | |
|---|---|---|---|---|---|---|---|
| C15:0 | 0,08 | 0,07 | 0,04 | 0,07 | 0,07 | 0,02 | 0.096 |
| **C16:0** | **27,19** | **26,27** | **6,15** | **22,41** | **21,10** | **4,74** | **<0.0001** |
| C18:0 | 6,91 | 6,63 | 1,35 | 6,48 | 6,21 | 1,43 | 0.100 |
| C20:0 | 0,20 | 0,17 | 0,12 | 0,17 | 0,16 | 0,07 | 0.476 |
| C22:0 | 0,55 | 0,50 | 0,29 | 0,41 | 0,38 | 0,14 | 0.021 |
| **C24:0** | **0,76** | **0,75** | **0,21** | **0,61** | **0,55** | **0,19** | **<0.0001** |
| **SFA** | **36,36** | **34,85** | **6,65** | **30,62** | **29,29** | **5,82** | **<0.0001** |
| **PUFA** | **36,33** | **36,77** | **5,35** | **42,08** | **42,15** | **4,76** | **<0.0001** |
| MUFA | 27,31 | 27,42 | 6,38 | 27,30 | 27,01 | 4,07 | 0.883 |
| OMEGA6/OMEGA3 | 0,18 | 0,17 | 0,08 | 0,17 | 0,16 | 0,06 | 0.908 |

**Table S1:** Composition of total plasma fatty acids in psoriasis compared to healthy individuals (expressed as % total fatty acids). P-values calculated with Mann-Whitney test. Bold values show p-values below the threshold after Bonferroni correction 0.05/23.

**Table S2:** Values of degree and betweenness centrality of the plasma fatty acids in psoriasis and healthy control group, used in the DSPC network analysis.

| PSORIASIS | | | | CONTROL | | | |
|---|---|---|---|---|---|---|---|
| Id | Label | Degree | Betweenness | Id | Label | Degree | Betweenness |
| C08323 | Nervonic acid | 11 | 63.48 | C00249 | Palmitic acid | 8 | 55.59 |
| C08281 | Behenic acid | 8 | 22.13 | C08320 | Tetracosanoic acid | 8 | 46.11 |
| C00712 | Oleic acid | 7 | 34.53 | C08323 | Nervonic acid | 8 | 21.91 |
| C06426 | gamma-Linolenic acid | 7 | 23.88 | C16537 | Pentadecenoic acid | 7 | 39.7 |
| C01530 | Stearic acid | 7 | 13.06 | C00219 | Arachidonic acid | 7 | 13.66 |
| C06425 | Arachidic acid | 6 | 23.74 | C06426 | gamma-Linolenic acid | 6 | 32.95 |
| C16537 | Pentadecenoic acid | 6 | 10.27 | C06427 | alpha-Linolenic acid | 5 | 37.41 |
| C08320 | Tetracosanoic acid | 6 | 7.62 | C00712 | Oleic acid | 5 | 7.63 |
| C00219 | Arachidonic acid | 5 | 21.95 | C01595 | Linoleic acid | 5 | 6.28 |
| C01595 | Linoleic acid | 5 | 6.71 | C08281 | Behenic acid | 5 | 2.24 |
| C06424 | Myristic acid | 5 | 1.03 | C06428 | Eicosapentaenoic acid | 4 | 4.09 |
| C08362 | Palmitoleic acid | 5 | 0.59 | C16526 | 11Z-Eicosenoic acid | 4 | 3.75 |
| C00249 | Palmitic acid | 4 | 16.58 | C01530 | Stearic acid | 4 | 1.67 |
| C06428 | Eicosapentaenoic acid | 3 | 1.67 | C06425 | Arachidic acid | 4 | 0 |
| C06427 | alpha-Linolenic acid | 3 | 0.83 | C08316 | Erucic acid | 3 | 8.62 |
| C14768 | 5,6-Epoxy-8,11,14-eicosatrienoic acid | 3 | 0 | C06424 | Myristic acid | 3 | 1.5 |
| C08322 | Myristoleic acid | 2 | 1.42 | C08362 | Palmitoleic acid | 3 | 1.13 |
| C08316 | Erucic acid | 2 | 0.5 | C08322 | Myristoleic acid | 3 | 1 |
| C03242 | Dihomo-gamma-linolenic acid | 2 | 0 | C06429 | Docosahexaenoic acid | 3 | 0 |
| C16526 | 11Z-Eicosenoic acid | 2 | 0 | C02679 | Dodecanoic acid | 2 | 1.75 |
| C06429 | Docosahexaenoic acid | 1 | 0 | C03242 | Dihomo-gamma-linolenic acid | 2 | 0 |
| | | | | C14768 | 5,6-Epoxy-8,11,14-eicosatrienoic acid | 1 | 0 |
